# Comprehensive exploration of tumor immune microenvironment feature and therapeutic response in colorectal cancer based on a novel immune-related long non-coding RNA prognostic signature

**DOI:** 10.3389/fgene.2022.962575

**Published:** 2022-08-25

**Authors:** Xueliang Zhou, Batuer Aikemu, Shuchun Li, Yanfei Shao, Hongtao Jia, Ling Huang, Hiju Hong, Sen Zhang, Qiushi Tang, Ruijun Pan, Jing Sun, Minhua Zheng

**Affiliations:** ^1^ Department of General Surgery, Ruijin Hospital, Shanghai Jiao Tong University School of Medicine, Shanghai, China; ^2^ Shanghai Minimally Invasive Surgery Center, Ruijin Hospital, Shanghai Jiao Tong University School of Medicine, Shanghai, China; ^3^ Shanghai Institute of Digestive Surgery, Ruijin Hospital, Shanghai Jiao Tong University School of Medicine, Shanghai, China; ^4^ Chinese Journal of Practical Surgery, China Medical University, Shenyang, China

**Keywords:** colorectal cancer, long non-coding RNA, immune signature, prognosis, tumor immune microenvironment, therapeutic response, candidate drugs, qRT-PCR

## Abstract

Colorectal cancer (CRC) is one of the most common malignant tumors with a high incidence rate and mortality. LncRNA is an important regulator of the immune system. It is of great significance to study immune-related lncRNAs (IR-lncRNAs) for CRC. In this study, we screened IR-lncRNAs differentially expressed in normal and CRC tissues, and Univariate Cox regression and the Least Absolute Shrinkage and Selection Operator were applied to construct IR-lncRNA prognostic signature in TCGA training dataset, and its predictive capability for the prognosis of CRC patients was verified in GSE39582 validation dataset. The novel signature was identified as an independent predictor of prognosis in CRC patients. In addition, the signature could accurately predict the feature of the immune microenvironment and therapeutic response in CRC patients. The CMap database was adopted to screen for small molecule candidate drugs that can reverse and treat high-risk CRC patients. Finally, the expression of six IR-lncRNAs were verified by qRT-PCR in clinical specimens from our patient cohort. In conclusion, we construct an IR-lncRNA prognostic signature, which is a powerful biomarker of CRC and can accurately predict the prognosis, immune microenvironment feature, and therapeutic response of CRC patients.

## Introduction

Colorectal cancer is one of the most common malignant tumors in the world. According to the global tumor epidemic Statistics (Globalcan 2020) released by the international agency for research on cancer (IARC) of the World Health Organization, it is estimated that there will be 1931600 new cases and 935,200 deaths of colorectal cancer in the world in 2020, ranking third and second among all malignant tumors respectively (Sung et al., 2021). The latest epidemiological survey shows that the incidence of the disease in males is more than that in females, and the age of onset is getting younger (Siegel et al., 2020). Although much remarkable progress has been made in early screening, surgical treatment, chemical therapy, and immunotherapy of colorectal cancer, which has improved the prognosis of colorectal cancer patients, the mortality is still high (Sumransub et al., 2021). At present, the clinical treatment and prognosis evaluation of colorectal cancer are mainly based on the TNM staging system. However, this evaluation method ignores the differences between individuals and the heterogeneity within the tumor and lacks the support of gene level (Lin et al., 2021). Molecular markers of CRC commonly used in clinical practice include RAS, BRAF and PI3KCA, which can assist in the diagnosis, staging, prognosis and clinical treatment of CRC. Studies have shown that patients with RAS mutation or BRAF mutation tend to have a poor prognosis (Jones et al., 2017; Venook et al., 2017). However, molecular typing of single genes has great limitations in accurate diagnosis and treatment of CRC. Therefore, The CRC Subtyping Consortium (CRCSC) proposed consensus Molecular subtype (CMS) typing based on gene expression in 2015 (Guinney et al., 2015). There are four types of CMS, including microsatellite stable (MSS) and immune activation type (CMS1), classic colorectal cancer type (CMS2), metabolic type (CMS3) and interstitial type (CMS4). CMS typing has been studied all over the world, but it has not been widely recognized and accepted, and its guiding role in clinical practice is limited (Valenzuela et al., 2021). Therefore, it is of great significance to explore accurate and reliable biomarkers to predict the prognosis for the individualized treatment of CRC patients.

Tumor microenvironment (TME) is defined as the local internal environment in the process of tumor genesis and development, which can provide a scaffold and barrier for the growth and metastasis of tumor cells, thus provide a “hotbed” for the occurrence and development of tumors (Chen et al., 2021a). The pathogenesis and development of colorectal cancer is a long-term and continuous pathological process, which has experienced stages from normal tissues, polyps, and adenomas to high-grade intraepithelial neoplasia, and then slowly transformed to malignant tumors. In this long transformation, the tumor immune microenvironment plays an extremely important role (Chen et al., 2021b). As an important component of TME, tumor immune microenvironment is regarded as the “seventh marker feature” of the tumor, which is mainly composed of immune cells (including lymphocytes, neutrophils, macrophages, etc.), structural components (such as fibroblasts, extracellular matrix, etc.), and intercellular communication-related molecules (chemokines, cytokines, growth factors, exosomes, etc.) (Chen et al., 2015). It is found that different degrees of immune cell infiltration in colorectal cancer tissues is closely related to the clinical stage of the tumor, suggesting that the local immune response status of tumor tissues can significantly affect the progression and clinical prognosis of colorectal cancer (Yoon et al., 2020). Therefore, the features of tumor immune microenvironment are increasingly considered as novel biomarkers affecting the development and prognosis of colorectal cancer.

A large number of studies have found that long non-coding RNA (lncRNA) plays an important regulatory role in tumor immune microenvironment of colorectal cancer, and can influence the differentiation, infiltration, and functional formation of immune cells (Yu et al., 2018). LncRNA is a functional RNA molecule that cannot be translated into proteins. LncRNA is usually long, ranging from 200 to 100000 nucleotides, which do not encode proteins and have mRNA-like structures. Their mechanisms of action are complex, diverse and play an important role in epigenetic, cell cycle, and cell differentiation regulation (Kopp and Mendell, 2018). LncRNA plays an important role in pre-transcriptional, transcriptional, and post-transcriptional levels. LncRNA can recruit chromatin remodeling complexes to mediate the silencing of some genes, act as decoys and bind to transcription factors to inhibit mRNA transcription, or act as sponges to absorb microRNA or directly bind to mRNA to degrade or inhibit mRNA translation (Bridges et al., 2021). Lnc-ITSN1-2 can promote the proliferation and activation of CD4^+^T cells and promote their differentiation into Th1/Th17 by targeting miR-125a and upregulating IL-23R (Nie and Zhao, 2020). Downregulation of lncRNA 2900052N01Rik (lnc-290) inhibits LPS-induced B cell proliferation, activation and differentiation by blocking LPS/TLR4 signaling pathway (Wang et al., 2021). At present, great progress has been made in the study of tumor immune microenvironment of colorectal cancer, but the role of lncRNAs in tumor immune microenvironment of CRC and its correlation with CRC prognosis and clinical characteristics have not been clarified. Therefore, it is of great value and significance to explore immune-related lncRNAs (IR-lncRNAs) as biomarkers to predict survival, tumor immune microenvironment feature and therapeutic response in colorectal cancer.

In this study, the Cancer Genome Atlas Project (TCGA) database was used to screen the key IR-lncRNAs in CRC, explore their role in the pathogenesis and development mechanism of CRC, and finally, develop a novel prognostic signature based on six IR-lncRNAs. Further research showed that this novel IR-lncRNA signature was not only a valid independent predictor of survival in CRC patients but also could accurately predict the feature of tumor immune microenvironment (immune cell infiltration, chemokines, immune and stromal scores, etc.) and therapeutic response (chemotherapy and immunotherapy). This novel signature was validated in GEO dataset, and the results were consistent with the prediction of the constructed prognostic model. Finally, the expression of six IR-lncRNAs were also verified by quantitative real-time PCR (qRT-PCR) in CRC clinical specimens.

## Materials and methods

### Collection of data and clinical information

In this study, the TCGA-COAD dataset was used as the discovery set. The gene expression profiles of 519 CRC patients (41 non-tumor samples and 478 tumor samples) and relevant clinical information were downloaded from TCGA (https://portal.gdc.cancer.gov/) (Colaprico et al., 2016). GSE39582 was downloaded from the GEO database (http://www.ncbi.nlm.nih.gov/geo)as an external validation set, containing a total of 585 CRC samples. The baseline information for all CRC individuals in TCGA and GSE39582 is presented in Table 1. LncRNA is identified by GTF files downloaded from Ensembl (http://asia.ensembl.org) for annotation and subsequent analysis. In addition, a list of immune-related genes (IRGs) can be obtained from the immune database (import) (https://www.immport.org/) (Bhattacharya et al., 2014) and the somatic mutation data file for CRC individuals is obtained from TCGA. The patients in the TCGA dataset and GSE39582 dataset do not receive any form of treatment before specimen collection.

**TABLE 1:** Clinical characteristics of CRC patients in TCGA and GSE39582 datasets.

Characteristics	TCGA dataset (N = 409)	GSE39582 dataset (N = 510)
Age		
<60	115	139
≥60	294	370
Unknown	0	1
Gender		
Male	224	275
Female	185	235
T		
Tis	1	3
T1	10	11
T2	70	41
T3	280	336
T4	48	99
Unknown	0	20
N		
N0	236	272
N1	98	120
N2	75	89
Unknown	0	29
M		
M0	301	442
M1	58	49
Unknown	50	19
Stage		
I	67	31
II	155	239
III	118	188
IV	58	48
Unknown	11	4
Status		
Alive	319	353
Dead	90	157

### Identification of differentially expressed IR-lncRNAs (DEIR-lncRNAs)

Pearson correlation analysis was conducted between lncRNAs and IRGs, and the correlation coefficient greater than 0.3 and *p* value less than 0.01 were considered immune-related lncRNAs (IR-lncRNAs). Differentially expressed genes (DEGs) were obtained using the R package “edgeR” for differential expression analysis of transcriptome data from the TCGA dataset (Robinson et al., 2010). DEIR-lncRNAs were identified by the intersection of DEGs and IR-lncRNAs. The threshold is set as |log2 Fold Change|>1 and false discovery rate<0.01.

### Establishment of IR-lncRNA prognostic signature and independent prognosis factor analysis

A total of 409 patients from the TCGA dataset with complete overall survival (OS) information were used as the training set to construct a prognostic model. First, Univariate Cox proportional risk regression was used to screen IR-lncRNAs (*p* < 0.05). The Least Absolute Shrinkage and Selection Operator (LASSO) is confirmed as a better method which can select the variables. This technique is a kind of compression estimation. By constructing a penalty function to obtain a relatively refined model, it compresses some regression coefficients and prevents the risk of overfitting, so as to obtain fewer and dominant effective variables. Therefore, based on Cox regression analysis, LASSO regression was used to further screen IR-lncRNAs associated with prognosis, and regression coefficients of each IR-lncRNA were obtained by the “glmnet” R package to establish CRC prognostic signature (Friedman et al., 2010). CRC individual riskscore is calculated as follows: riskscore = expression of lncRNA a × regression coefficient of lncRNA a + expression of lncRNA b × regression coefficient of lncRNA b +……+ expression of lncRNA n × regression coefficient of lncRNA n (Chen et al., 2017). Then, taking the median value of the riskscore in the training group as the risk threshold, all CRC individuals in the training set and validation set were divided into the high-risk group and the low-risk group. We performed the univariate and multivariate regression analysis of riskscore based on IR-lncRNA signature to determine whether the riskscore could be an independent prognostic factor for CRC individuals. Principal component analysis (PCA), Kaplan -Meier survival curve, receiver operating characteristic curve (ROC) were used to evaluate the predictive capability of riskscore for CRC individual’s prognosis in training set and validation set. The Kaplan-Meier survival curves of single IR-lncRNA in prognostic signature were also constructed in TCGA training set and GEO external validation set. Finally, we evaluated the prognosis predictive capability of the riskscore for each of the individuals with CRC in various clinical subtypes and investigated the association between the clinical characteristics and the riskscore.

### Construction of clinical nomogram prediction model with IR-lncRNA signature

Age, gender, T stage, N stage, M stage, and riskscore were included in the prognostic analysis. The prognostic nomogram model was established by using the “rms” R package in the TCGA dataset and GSE39582 dataset respectively, and the overall survival (OS) of 1, 3, and 5 years of CRC individuals were predicted respectively. Multivariate Cox regression analysis was used to verify the results, and calibration function was used to draw the calibration curve for predicting the OS rate at 1-, 3- and 5-years to evaluate the prediction consistency of nomogram. Finally, the ROC curves of the nomogram for predicting the OS rate of patients were constructed in the TCGA dataset and GSE39582 dataset respectively, so as to evaluate the prediction accuracy of the nomogram.

### Mutation analysis

The mutation annotation file (MAF) of TCGA-COAD mutation data was downloaded from the TCGA database (https://portal.gdc.cancer.gov/). The mutation data were analyzed by maftools, and the tumor mutation burden (TMB) was calculated (Mayakonda et al., 2018).

### Screening of candidate drugs

Connectivity map (CMap; https://clue.io/) database is a valuable database in the field of pharmacogenomics. The database collects genome-wide transcriptional expression data of cultured human cells processed with bioactive small molecules and simple pattern matching algorithm. Based on the differentially expressed genes between the high-risk and low-risk groups, we uploaded them to identify related small molecule compounds. Enrichment fraction close to -100 indicates that the small molecule compounds have antagonistic effect on these DEGs and is a candidate drug for the reversal and treatment of high-risk CRC patients. Finally, the 3D structures of these small molecule compounds were obtained from the PubChem database (https://pubchem.ncbi.nlm.nih.gov/).

### Exploration of immune microenvironment features and immunotherapy sensitivity evaluation in CRC individuals

The “CIBERSORT” R package is a novel deconvolution algorithm to estimate the proportion of immune cells based on RNA-Seq data and microarray expression data. We calculated the proportion of 22 immune cells in the immune microenvironment of each tumor sample in the TCGA dataset and GSE39582 dataset by the “CIBERSORT” R package (Newman et al., 2015). The “ESTIMATE” R package can calculate the immune and stromal scores of each sample through RNA-Seq data, and then evaluate the purity of the tumor. There are 4 scoring indicators: immune score, stromal score, ESTIMATE score and tumor purity (Kang et al., 2020). We used the “ESTIMATE” package to compare immune score, stromal score, and tumor purity in the high-risk and the low-risk groups. The cytolytic score is an important index to evaluate the function of cytotoxic immune cells. It is mainly calculated by the average expression of five granzymes (GZMA, GZMK, GZMM, GZMB, GZMH) and perforin-1 (PRF1) (Rooney et al., 2015). We also compared the expression of chemokine, chemokine receptor, and human leukocyte antigen in the high-risk group and the low-risk group in the TCGA dataset and GSE39582 dataset to explore the features of tumor immune microenvironment of CRC individuals.

We compared the expression levels of tumor immune checkpoints (ICs) in the high-risk and low-risk groups, and analyzed the correlation between the novel signature and the expression of ICs, to predict the sensitivity of immunotherapy. Previous studies have shown that Mismatch Repair (MMR) status (dMMR and pMMR) and TMB are important markers for predicting sensitivity of immune checkpoint inhibitors (Picard et al., 2020). Therefore, we compared TMB in the high-risk and the low-risk groups in the TCGA dataset. We also analyzed the relationship between MMR status and the riskscore by investigating the expression levels of four MMR proteins (MLH1, MSH2, MSH6, PMS2) in both high and low risk groups.

### Single-sample gene set enrichment analysis (ssGSEA)

ssGSEA is an enrichment analysis for a single sample, which can be used to evaluate the infiltration degree of immune cells in the tumor microenvironment. We adopted the “GSVA” R package to evaluate the infiltration degree of 28 types of immune cells in each CRC sample (Yu et al., 2020), which were divided into tumor-promoting and tumor-inhibiting types. The correlation between tumor-promoting and tumor-inhibiting activity in the immune microenvironment of each sample was also calculated.

### Prediction of therapeutic response

IC50 refers to the drug concentration when the growth is inhibited by 50%, which is an important indicator of drug sensitivity. The “pRRophetic” R package is used to calculate the IC50 of common chemotherapeutic drugs for CRC (Paul et al, 2014). GSE16066, GSE19862, GSE28702, and GSE62080 downloaded from the GEO database were used to verify the capability of the novel signature to predict therapeutic response.

### Functional enrichment analysis

We used the “clusterProfiler” R package to enrich and analyze Gene Ontology (GO) and Kyoto Encyclopedia of Genes and Genomes (KEGG) in R software (Yu et al., 2012). *p* < 0.05 was considered statistically significant, and the “ggplot2″ R package was used for visual analysis (Ito and Murphy, 2013).

### Expression validation of the IR-lncRNA signature in CRC clinical specimens

The UALCAN database was employed to verify the expression level of the six IR-lncRNAs in CRC tumor and non-tumor samples (Chandrashekar et al., 2017). Furthermore, 36 matched tumor and non-tumor tissue specimens were collected from Ruijin Hospital, Shanghai Jiao Tong University School of Medicine after approved by the local ethics committee of Ruijin Hospital (No. 2020-384). The patients did not receive any form of treatment before specimen collection. The total RNA of tissue specimens was extracted with RNA isolater (Vazyme, China), and then, one μ g of total RNA was reverse transcribed into cDNA with Hiscript III RT Supermix and gDNA wiper (Vazyme, China). ChamQ universal SYBR qPCR Master Mix (Vazyme, China) was used for real-time fluorescence quantitative PCR (qRT-PCR) analysis, and the expression level of GAPDH served as an internal control. The cycle scheme is 95°C for 5 min, 40 cycles at 95°C for 15 s, 60°C for 60 s, 72°C for 5 min. All primers were synthesized by Tsingke (Beijing, Shanghai) company and primer sequences are presented in Table 2. The comparative Ct (2^−ΔΔCt^) method was adopted to calculate the relative expression level of the six IR-lncRNAs.

**TABLE 2:** Primer sequences used in PCR.

Symbol	Sequence
CATIP-AS1	Forward primer: CCC​TAC​GGG​TGT​GCA​TTT​CA; Reversed primer: ACC​ACA​CTG​GGG​GTT​AGA​GA
GABPB1-AS1	Forward primer: AAA​TCG​CGG​GGA​TGA​TCT​GG; Reversed primer: CCA​CCG​GAT​GTG​GAA​GTT​GA
MMP25-AS1	Forward primer: GGC​TCA​TTT​CAT​CGG​CAA​GC; Reversed primer: TTG​TCT​CAG​GTT​CCT​CGC​CT
PCAT6	Forward primer: CTT​CGC​CCC​TAG​ATA​CAC​CC; Reversed primer: GGT​GGT​GGT​AGA​AGC​ACG​AG
NSMCE1-DT	Forward primer: TGG​AAG​CTT​TGA​TGG​TGG​TCA; Reversed primer: GAC​TTA​GGC​CAT​GGA​TGG​GT
TSPEAR-AS2	Forward primer: CAA​ACA​TCC​GGG​GAC​CCT​TA; Reversed primer: TTG​AGA​GCA​GAG​TCG​CGC​A
GAPDH	Forward primer: TGA​AGG​TCG​GAG​TCA​ACG​G; Reversed primer: CCT​GGA​AGA​TGG​TGA​TGG​G

### Statistical analysis

All statistical analyses were performed by R Software (Version 4.1.0) and GraphPad Prism 6.0 (GraphPad Inc., San Diego, CA, United States). The *p* value of all statistical data were tested by bilateral statistical test, *p* < 0.05 was considered statistically significant. Wilcoxon rank-sum test was used to compare differences between the two groups. K-W test was used to compare three or more groups. Kaplan-Meier analysis was used to assess survival differences between the low-risk and the high-risk groups.

## Results

### Identification of DEIR-lncRNAs

**FIGURE 1:**
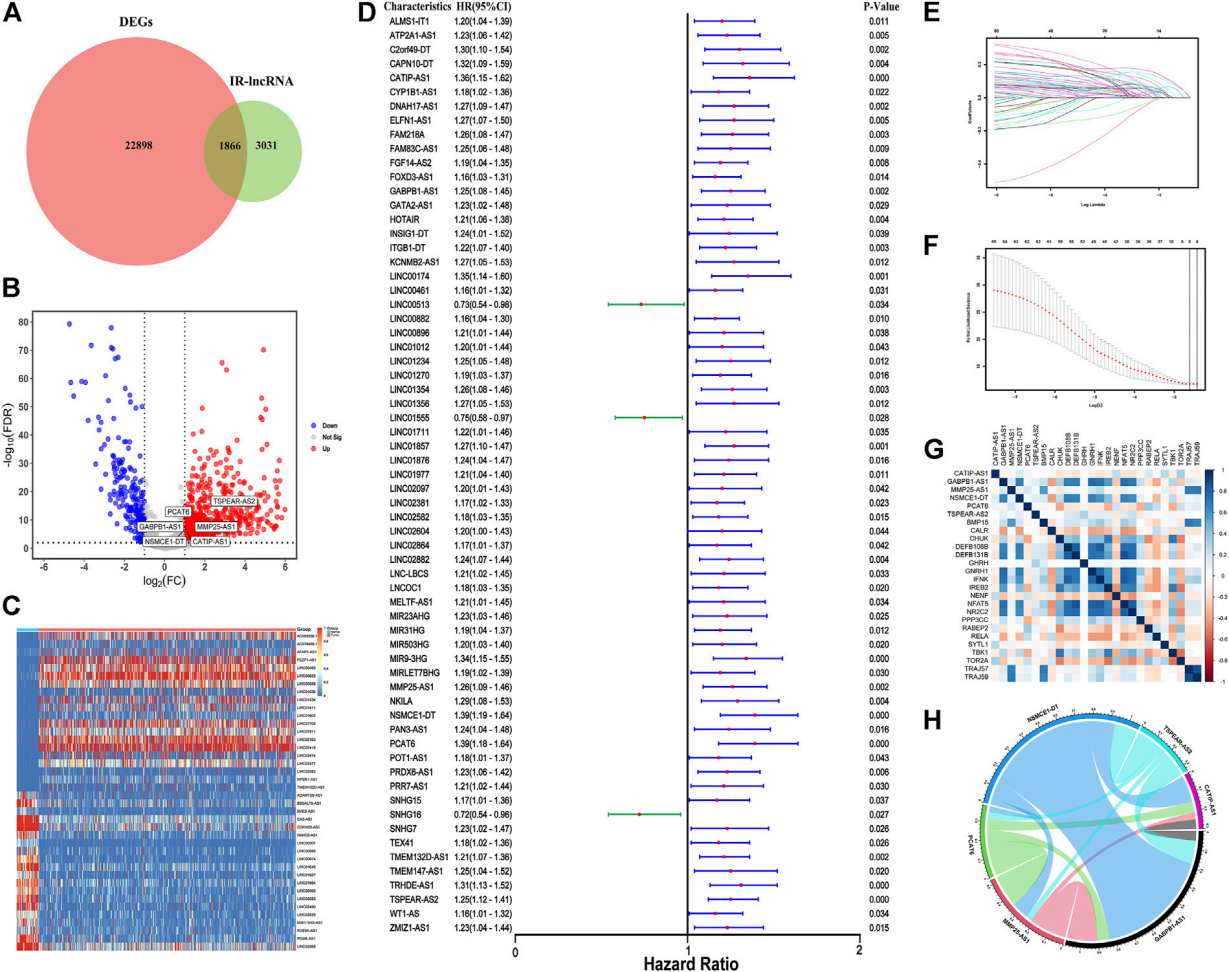
Construction of IR-lncRNA prognostic signature. **(A)** Venn diagram to show the intersection of IR-lncRNAs and DEGs; **(B)** Volcano plot of the DEIR-lncRNAs between CRC normal and tumor samples; **(C)** Heatmap to show the difference in the expression of the top 20 and low 20 DEIR-lncRNAs in normal and tumor samples from the TCGA dataset; **(D)** Forest plot to show the results of the univariate cox regression analysis between DEIR-lncRNAs and prognosis; **(E–F)** LASSO-Cox regression analysis to screen the prognostic signature; **(G)** Correlation between six IR-lncRNAs and IRGs; **(H)** Correlation chord diagram of the six IR-lncRNAs.

The process of this study is shown in Supplementary Figure S1. First, we identified 22898 DEGs in 41 normal and 478 tumor samples in the TCGA-COAD dataset. LncRNAs were annotated according to the GTF file downloaded from Ensembl, and co-expression was conducted between the known IRGs from the import database and lncRNAs. A total of 4897 IR-lncRNAs were identified. Venn diagram showed the intersection of IR-lncRNAs and DEGs, including 1866 DEIR-lncRNAs (Figure 1A). The volcano plot showed 1866 DEIR-lncRNAs between CRC normal and tumor samples and the expression of six IR-lncRNAs were significantly up-regulated in tumor samples (Figure 1B). The Heatmap showed the difference in the expression of the top 20 and low 20 DEIR-lncRNAs in normal and tumor samples (Figure 1C).

### Construction of IR-lncRNA prognostic signature

67 OS-related IR-lncRNAs were screened by univariate cox regression analysis, of which LINC00513, LINC01555, and SNHG16 were the protective factors of CRC, and CATIP-AS1, GABPB1-AS1, PCAT6 and other lncRNAs were the risk factors of CRC (Figure 1D). The optimal lambda value was obtained by LASSO Cox regression and cross-validation. Finally, six IR-lncRNAs were screened to construct the prognostic signature (Figures 1E,F). The full names and coefficients of the six lncRNAs are shown in Table 3. The riskscore based on the signature of these six IR-lncRNAs can be used to predict the prognosis of CRC individuals. The calculation formula of riskscore is as follows: 0.0322 **×** expression of CATIP-AS1 + 0.0073 **×** expression of GABPB1-AS1 + 0.0041**×** expression of MMP25-AS1 + 0.0625 **×** expression of NSMCE1-DT + 0.0990 **×** expression of PCAT6 + 0.0870 **×** expression of TSPEAR-AS2. We also analyzed the correlation between six IR-lncRNAs and IRGs, the results showed that six IR-lncRNAs were significantly associated with IRGs (Figure 1G). The correlation of the six IR-lncRNAs was represented by a chord diagram (Figure 1H).

**TABLE 3:** The information of six IR-lncRNAs.

IR-lncRNA	Full name	Coefficient
CATIP-AS1	Ciliogenesis Associated TTC17 Interacting Protein Antisense RNA 1	0.0322
GABPB1-AS1	GA Binding Protein Transcription Factor Subunit Beta 1 Antisense RNA 1	0.0073
MMP25-AS1	Matrix Metallopeptidase 25 Antisense RNA 1	0.0041
NSMCE1-DT	NSE1 Homolog, SMC5-SMC6 Complex Component Divergent Transcript	0.0625
PCAT6	Prostate Cancer Associated Transcript 6	0.0990
TSPEAR-AS2	Thrombospondin Type Laminin G Domain and EAR Repeats Antisense RNA 2	0.0870

### Evaluation and verification of the IR-lncRNA signature

**FIGURE 2:**
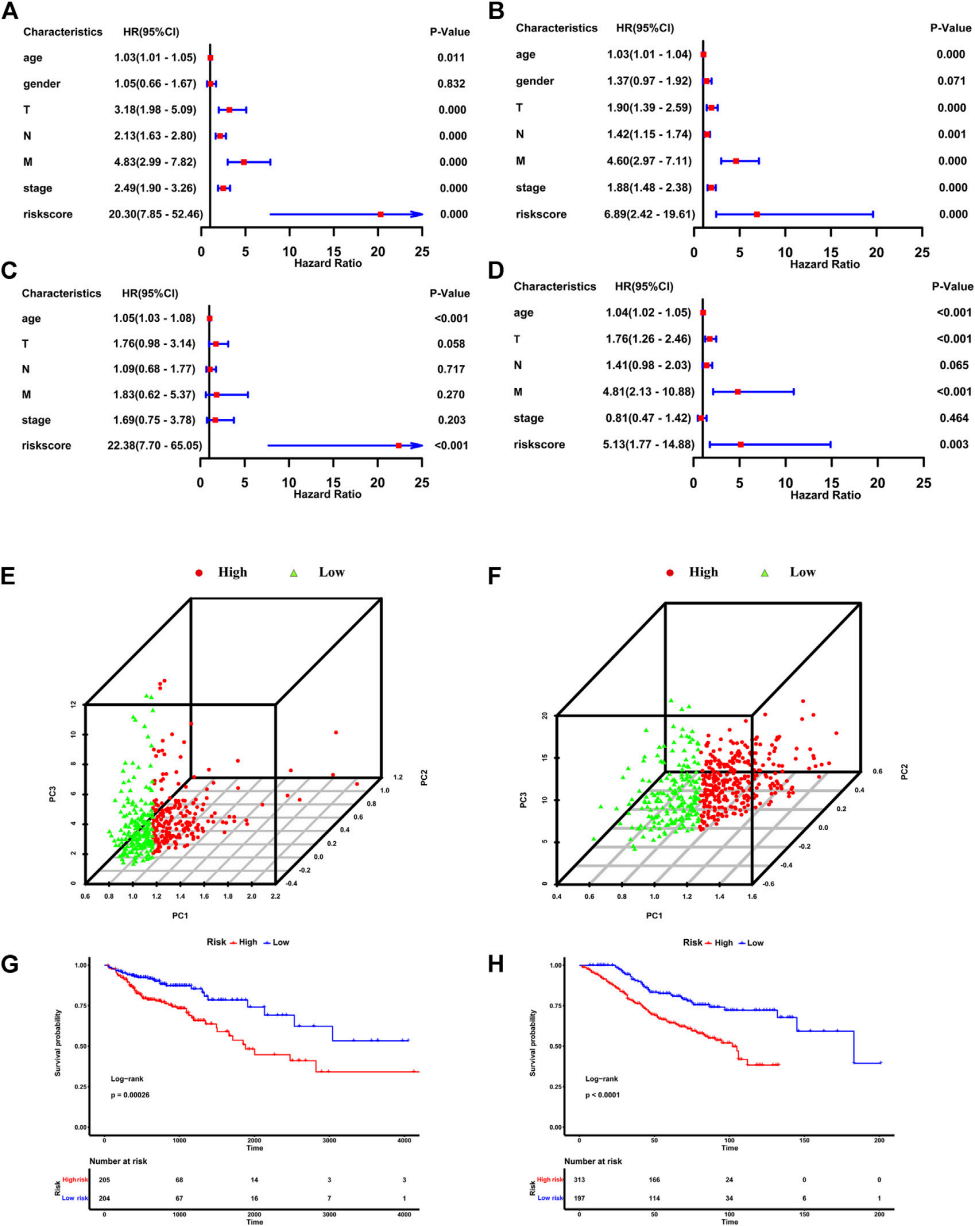
The IR-lncRNA signature is the independent risk factor for the prognosis of CRC. Results of the univariate and multivariate Cox regression analyses regarding OS in the TCGA **(A,C)** and the GSE39582 **(B,D)** datasets; PCA analysis to distinguish high-risk and low-risk groups in TCGA **(E)** and GSE39582 **(F)** datasets; Kaplan-Meier analysis to show the OS of patients in high-risk and low-risk groups in the TCGA **(G)** and GSE39582 **(H)** datasets.

In the TCGA dataset and GSE39582 dataset, CRC individuals were divided into the high-risk group (with high riskscore) and the low-risk group (with low riskscore) according to the median value of riskscore calculated according to the above formula. Univariate Cox regression and multivariate cox regression analyses were used to determine whether the riskscore based on six IR-lncRNAs was an independent risk factor for predicting the prognosis of CRC in the TCGA training set and GSE39582 validation set. Univariate cox regression analysis showed that age, T stage, N stage, M stage and TNM stage were significantly correlated with OS of the TCGA training set (*p* < 0.05) (Figure 2A). Multivariate regression analysis showed that the riskscore was an independent risk factor for OS of the TCGA training set (HR: 22.38,95% CI = 7.70-65.05, *p* < 0.05) (Figure 2C). This result was verified in the GSE39582 validation set (Figures 2B,D). PCA analysis showed that the riskscore could well distinguish the high-risk and low-risk groups (Figures 2E,F). Kaplan-Meier analysis further showed that the OS of patients in the high-risk group was significantly shorter than that in the low-risk group, indicating that the riskscore was an important index to predict the prognosis of CRC (Figures 2G,H).

**FIGURE 3:**
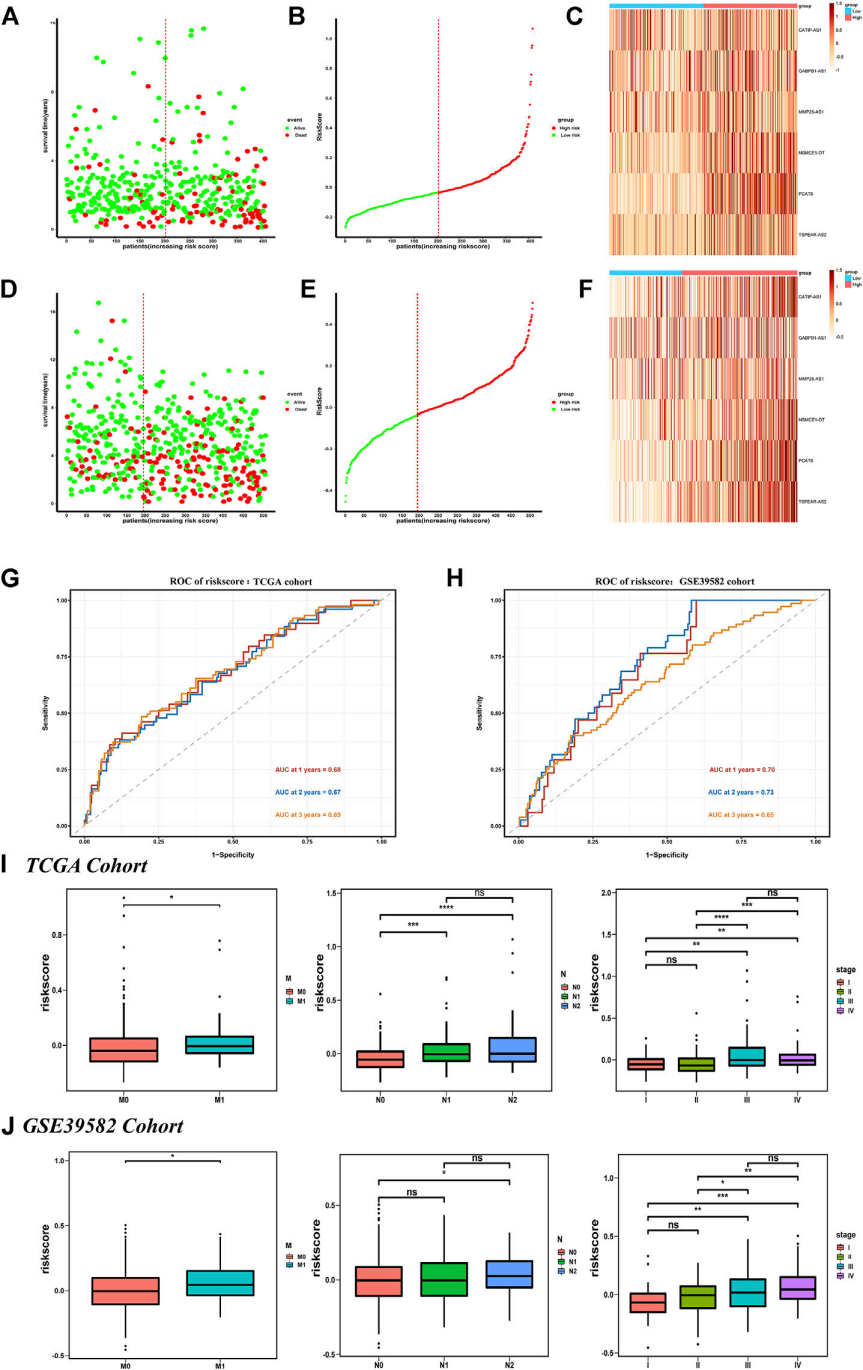
Prognostic value of the IR-lncRNA signature. The survival status plots, riskscore distribution plots and the heatmap of six prognostic IR-lncRNAs in the TCGA **(A–C)** and GSE39582 **(D–F)** datasets; AUC of time-dependent ROC curves for the riskscore in the TCGA **(G)** and GSE39582 **(H)** datasets; The relationship between the riskscore and the clinicopathological features (M, N and stage) in the TCGA **(I)** and the GSE39582 **(J)** datasets. ^*^ *p* < 0.05, ^**^ *p* < 0.01, ^***^ *p* < 0.001, ^****^ *p* < 0.0001. ns, no significance.

In the TCGA training set, the survival status plot showed that in the high-risk group, the survival time of CRC individuals was significantly shorter than that in the low-risk group, and their prognosis is generally poor (Figure 3A). The riskscore distribution plot showed the distribution of riskscore (Figure 3B). The heatmap showed the expression patterns of the six IR-lncRNAs. The expression of IR-lncRNA in the high-risk group was significantly higher than that of the low-risk group (Figure 3C). Similarly, this result was verified in the GSE39582 dataset (Figures 3D–F). The ROC curves for predicting the survival rate of 1-year, 2-years, and 3-years in the TCGA training set (Figure 3G) and GSE39582 validation set (Figure 3H) showed that the signature of six IR-lncRNAs could effectively evaluate the prognosis of CRC individuals.

We found that the novel signature was closely related to the clinicopathological features of CRC individuals and can be widely used in CRC populations with different clinicopathological characteristics. The results showed that among the subgroups stratified according to the clinicopathological features in the TCGA dataset, the prognosis of the low-risk group was significantly better than that of the high-risk group, and the survival rate of individuals in the low-risk group was higher (*p* < 0.05) (Supplementary Figure S2A). It was also verified in the GSE39582 dataset (Supplementary Figure S2B), there were significant differences in riskscore based on six IR-lncRNAs in N stage, M stage, and TNM stage (*p* < 0.05) in the TCGA (Figure 3I) and GSE39582 (Figure 3J) datasets.

### Establishment of a nomogram based on the novel signature

**FIGURE 4:**
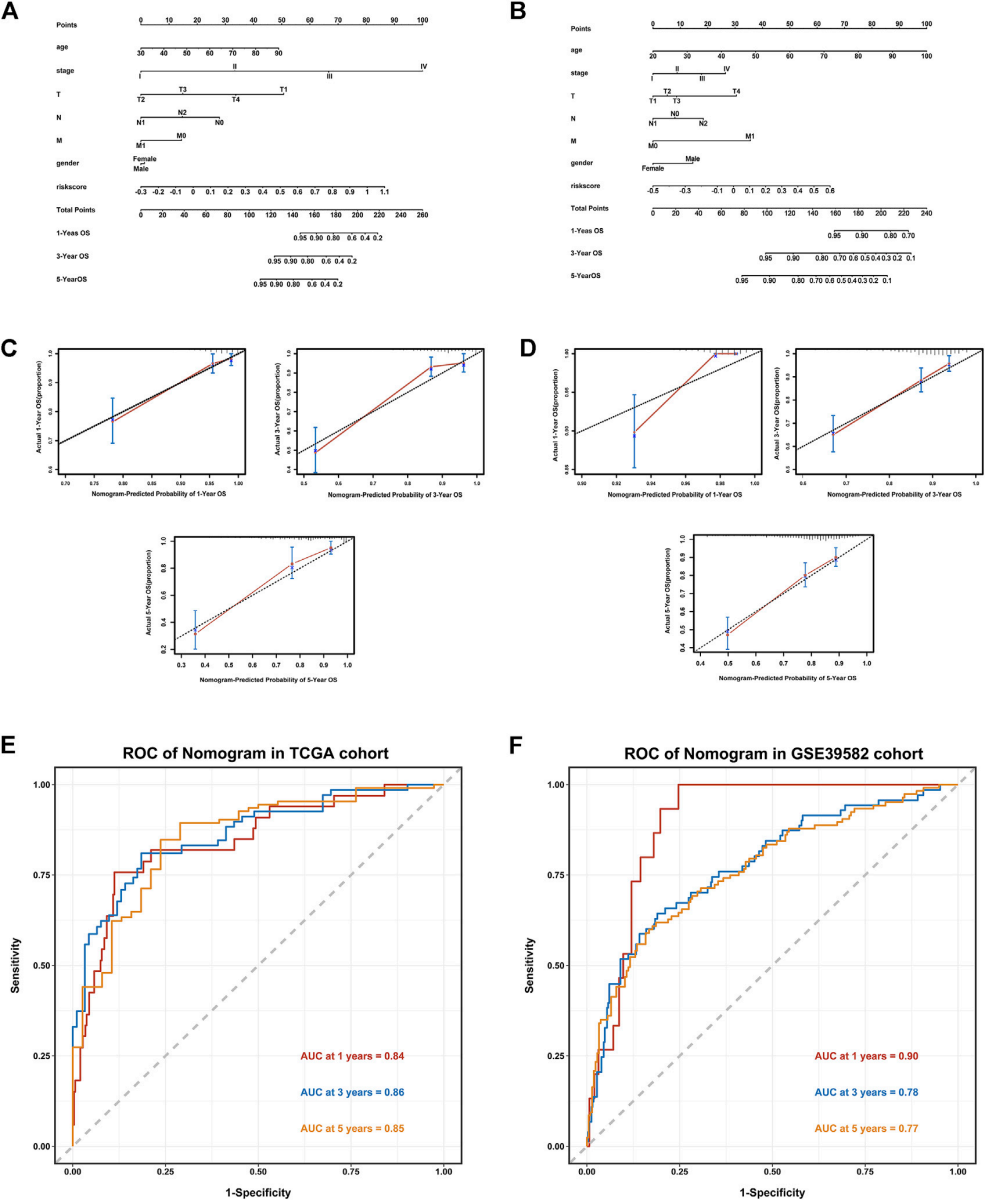
Establishment of a Nomogram based on the IR-lncRNA signature. Nomograms for predicting 1-year,3-years and 5-years OS in the TCGA **(A)** and GSE39582 **(B)** datasets; Calibration curves for the nomogram predicting 1-,3- and 5-years OS in the TCGA **(C)** and GSE39582 **(D)** datasets; AUC of time-dependent ROC curves for the nomograms in the TCGA **(E)** and GSE39582 **(F)** datasets.

The clinicopathological features such as age, gender, T stage, N stage, M stage, and riskscore were included in Nomogram, and the prognostic models based on the signature of the TCGA training set (Figure 4A) and the GSE39582 validation set (Figure 4B) were constructed. The calibration curve showed that the prediction effect of 1-, 3- and 5-years survival rate of CRC individuals indicate an excellent capability (Figures 4C,D). The ROC curve showed that the accuracy of the model for predicting the 1-, 3-, and 5-years survival rate was high (Figures 4E,F).

### Mutation analysis based on the novel signature

**FIGURE 5:**
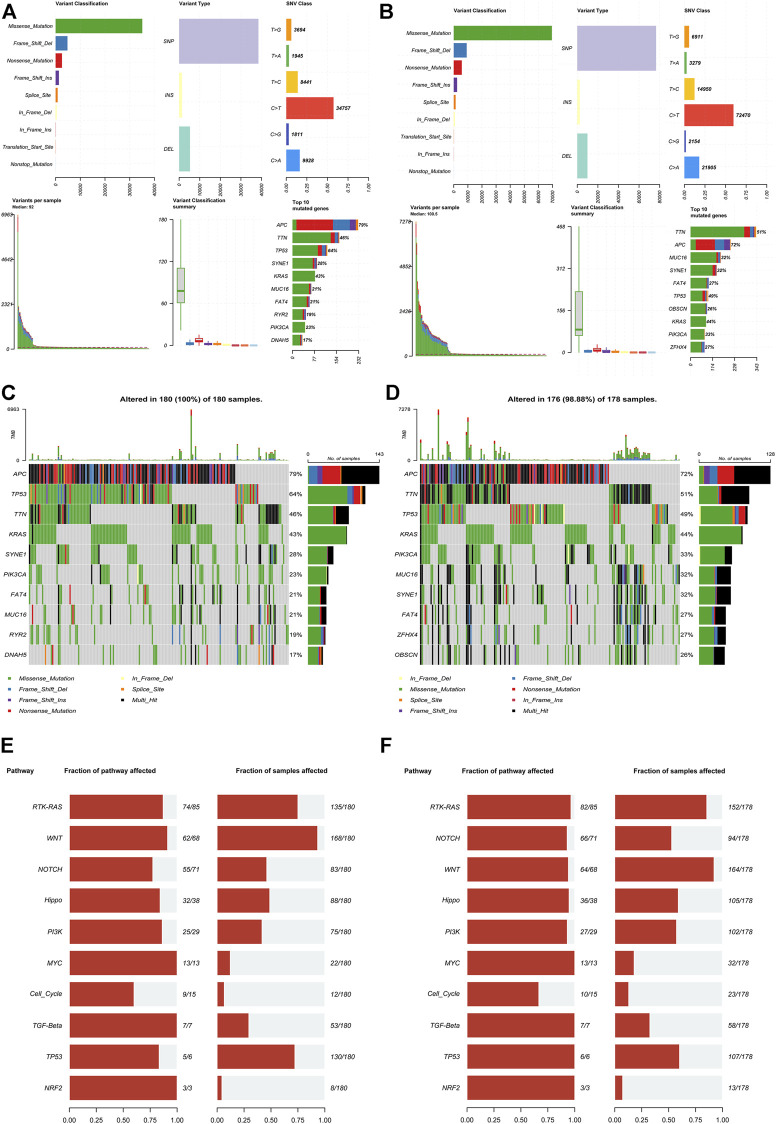
Mutation analysis based on the IR-lncRNA signature. MAF-summary plots, oncoplots and oncogenic pathways of the somatic mutation between the high-risk **(A,C,E)** and low-risk **(B,D,F)** groups in the TCGA dataset.

We downloaded and analyzed the gene mutation annotation file (MAF) in the TCGA dataset to analyze the difference of gene mutation between the high-risk group and the low-risk group according to the signature. The MAF-summary plots of gene mutation in the high-risk group (Figure 5A) and low-risk group (Figure 5B) were shown in the figure. The oncoplots showed that APC(79%), TP53 (64%), TTN (46%), KRAS (43%), SYNE1(28%) and PIK3CA(23%) were the top six genes with the highest mutation frequency in the high-risk group (Figure 5C), while APC(72%), TTN (51%), TP53 (49%), KRAS (44%), PIK3CA(33%) and MUC16(32%) were the top six genes with the highest mutation frequency in the low-risk group (Figure 5D). TP53 was relatively high-mutated while PIK3CA was relatively low-mutated in the high-risk group. The mutant genes in the high-risk group and low-risk group were significantly enriched in RTK-RAS, WNT and NOTCH signaling pathways (Figures 5E,F).

### The exploration of tumor immune microenvironment feature and immunotherapy sensitivity based on IR-lncRNA signature in CRC individual

**FIGURE 6:**
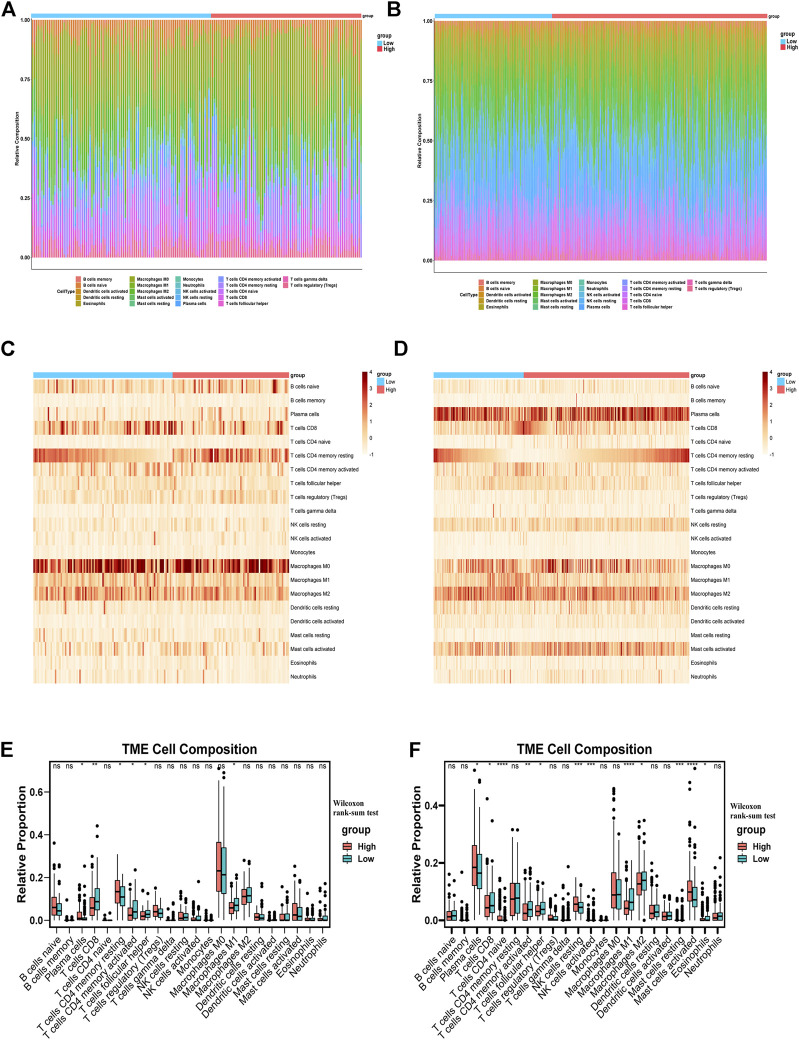
The distribution of 22 tumor-infiltrating immune cells between the high-risk and low-risk groups. The barplots, heatmaps and boxplots of 22 tumor-infiltrating immune cells distribution between the high-risk and low-risk groups in the TCGA **(A,C,E)** and GSE39582 **(B,D,F)** datasets. ^*^ *p* < 0.05, ^**^ *p* < 0.01, ^***^ *p* < 0.001, ^****^ *p* < 0.0001. Ns, no significance.

**FIGURE 7:**
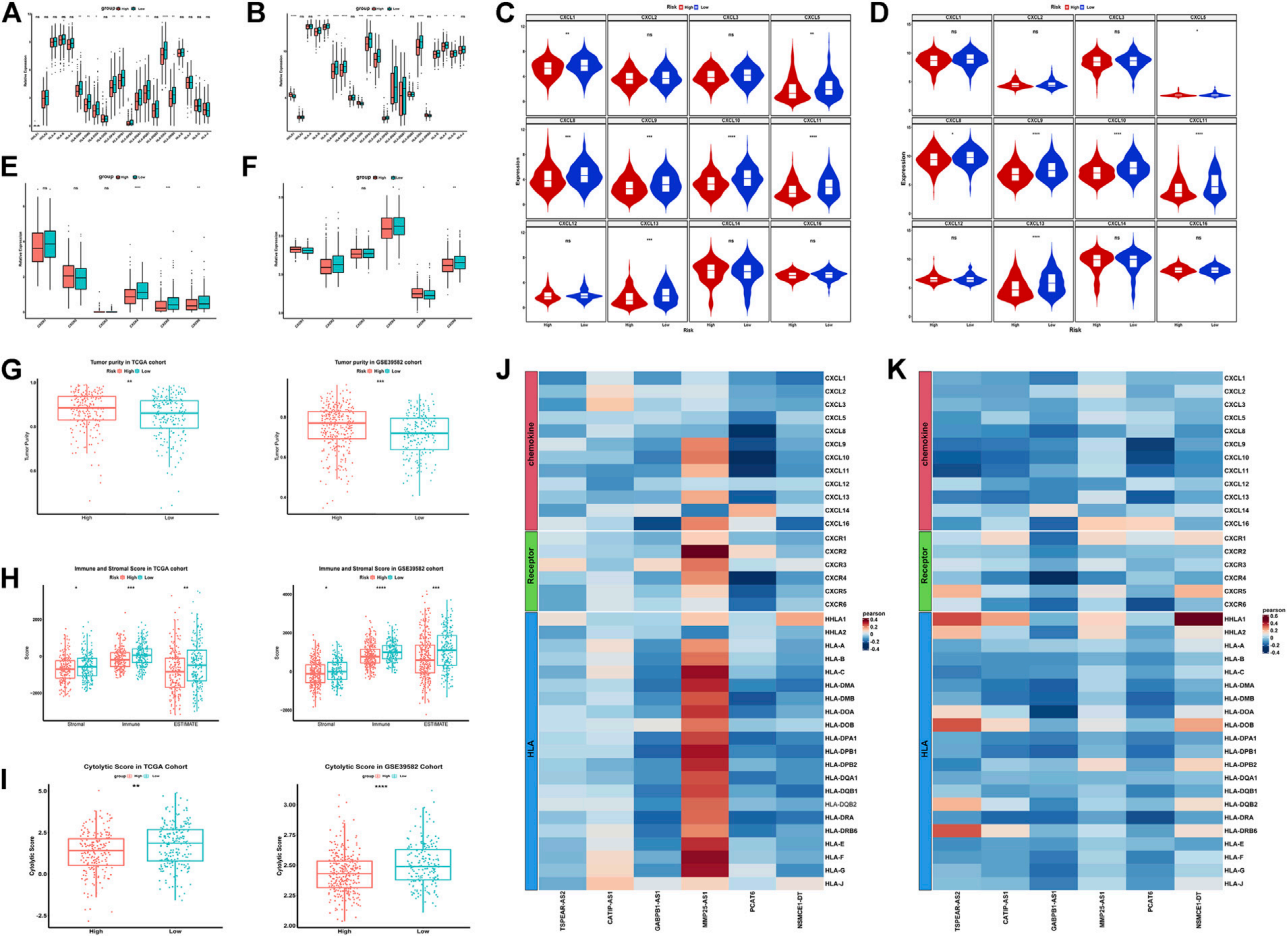
Exploration of the features of immune microenvironment between the high-risk and low-risk groups in colorectal cancer. The expression of HLA gene family between the high-risk and low-risk groups in the TCGA **(A)** and GSE39582 **(B)** datasets; The expression of CXC chemokine family between the high-risk and low-risk groups in the TCGA **(C)** and GSE39582 **(D)** datasets; The expression of CXCR receptor family between the high-risk and low-risk groups in the TCGA **(E)** and GSE39582 **(F)** datasets; The tumor purity, immune and stromal score and the cytolytic score between the high-risk and low-risk groups in the TCGA and GSE39582 datasets **(G–I)**; The correlation between six IR-lncRNAs and HLA gene, CXC chemokine and CXCR receptor in TCGA **(J)** and GSE39582 **(K)** datasets. ^*^ *p* < 0.05, ^**^ *p* < 0.01, ^***^ *p* < 0.001, ^****^ *p* < 0.0001. Ns, no significance.

In order to study the different features of immune microenvironment between the high-risk group and the low-risk group, based on CIBERSORT, we analyzed and compared the scores of 22 kinds of immune cell infiltration between the high-risk and the low-risk groups in the TCGA training set and the GSE39582 validation set. The columnar accumulation diagram showed the whole landscape of 22 kinds of immune cell infiltration in CRC individuals (Figures 6A,B). The heatmap showed the differences of 22 kinds of immune cell infiltration in different CRC individuals (Figures 6C,D). The results of CIBERSORT analysis showed that plasma cell, CD8+T cell, Macrophages M1, follicular helper T cell and CD4 memory activated T cell were significantly different between the high-risk and the low-risk groups in the training set and validation set. Plasma cell was significantly up-regulated in the high-risk group, while CD8^+^ T cell, macrophages M1, follicular helper T cell, and CD4 memory activated T cell were significantly up-regulated in the low-risk group (Figures 6E,F). Based on CIBERSORT, we analyzed the correlation between the six IR-lncRNAs and the above five immune infiltrating cells, and of which PCAT6 was the most closely related to immune cell infiltration (Supplementary Figure S3). The expression of HLA gene family, CXC chemokine family, CXCR receptor family between the high-risk and low-risk groups were analyzed in the TCGA (Figures 7A,C,E) and GSE39582 (Figures 7B,D,F) datasets. Through the “ ESTIMATE” package, we found that the tumor purity of tissues in the high-risk group was significantly higher than that in the low-risk group (Figure 7G), and the immune and stromal scores of individuals in the high-risk group were significantly lower than those in the low-risk group in the TCGA and GSE39582 datasets (Figure 7H). At the same time, the cytolytic score of immune cells in the high-risk group was significantly lower than that in the low-risk group (Figure 7I). We also analyzed the correlation between six IR-lncRNAs and HLA gene family, CXC chemokine family, CXCR receptor family in the TCGA (Figure 7J) and GSE39582 (Figure 7K) datasets. In addition, we conducted ssGSEA analysis of 28 immune cells in the training set and validation set, and the boxplot showed the differences of 28 immune cell infiltration in high-risk and low-risk groups (Supplementary Figure S4A,B). The heatmap showed the infiltration differences of 28 immune cells in CRC individuals (Supplementary Figure S4C,D). Finally, we divided 28 types of immune infiltrating cells based on ssGSEA into “Anti-tumor and Immune Activation” and “Pro-tumor and Immune Suppression” categories and analyzed the correlation between the two types of immune cell infiltration in the training set and validation set. The results showed that in the tumor microenvironment, immune activation and immune suppression existed at the same time, there was a feedback effect between each other, and the two promoted and inhibited each other (Supplementary Figure S4E,F).

**FIGURE 8:**
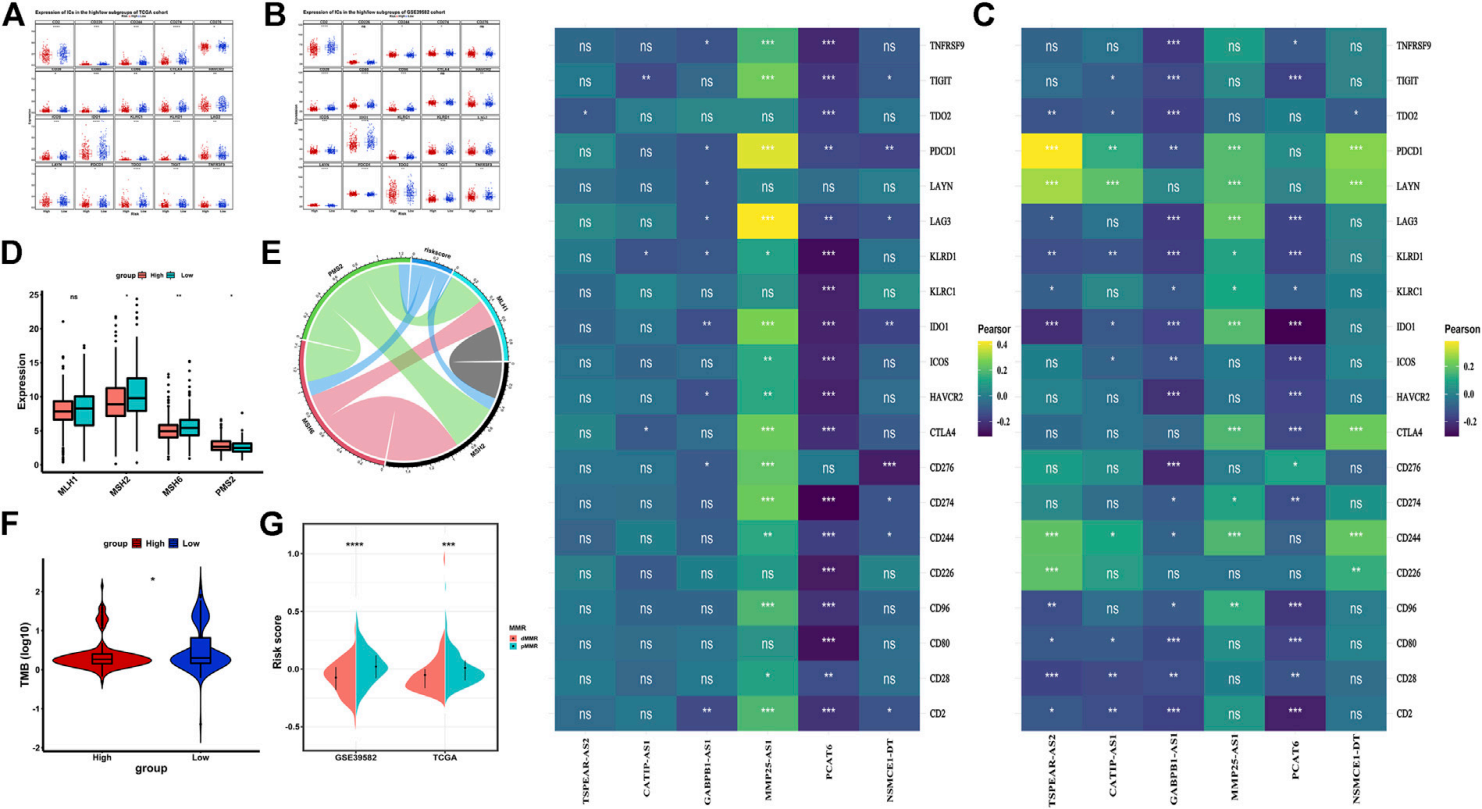
Immunotherapy sensitivity in the high-risk and low-risk groups in colorectal cancer. **(A)** The expression of Immune checkpoints between the high-risk and low-risk groups in the TCGA and GSE39582 datasets; The correlation between six IR-lncRNAs and immune checkpoints in the TCGA **(B)** and GSE39582 **(C)** datasets; **(D)** The expression of mismatch repair genes (MLH1, MSH2, MSH6, PMS2) between the high-risk and low-risk groups in the TCGA dataset; **(E)**The Correlation analysis of mismatch repair genes (MLH1, MSH2, MSH6, PMS2); **(F)** The comparison of tumor mutation burden (TMB) between the high-risk and low-risk groups in the TCGA dataset; **(G)** The comparison of mismatch repair status between the high-risk and low-risk groups in the TCGA and GSE39582 datasets. ^*^ *p* < 0.05, ^**^ *p* < 0.01, ^***^ *p* < 0.001, ^****^ *p* < 0.0001. Ns, no significance.

Immune checkpoint inhibitor is an important treatment option for CRC. Tumor immune checkpoint (IC) is an important factor influencing the effect of tumor immunotherapy. Generally speaking, the higher the expression of ICs, the higher the sensitivity of immunosuppressant therapy, and the better the effect of immunotherapy. We analyzed the expression of 20 ICs between the high-risk and the low-risk groups in the TCGA dataset and the GSE39582 dataset. The results showed that most ICs in the low-risk group had higher expression and better effect of immunotherapy (Figure 8A). Then, we showed the correlation between the six IR-lncRNAs and 20 ICs in the TCGA (Figure 8B) and GSE39582 (Figure 8C) datasets. Previous studies have shown that Microsatellite Stability (MSI)/Mismatch Repair (MMR) and Tumor Mutation Burden (TMB) are important biomarkers of CRC immunotherapy sensitivity. MSI-H/dMMR status and higher TMB score suggest that immunotherapy is more sensitive. At present, the clinical detection of MSI/MMR mainly detects the expression of four MMR proteins (MLH1, MSH2, MSH6, and PMS2). We analyzed the expression of four MMR proteins in the high-risk group and the low-risk group of the TCGA dataset (Figure 8D) and analyzed their correlation with riskscore (Figure 8E). Further analysis showed that in the TCGA training set and GSE39582 validation set, the TMB score of the low-risk group was higher than that of the high-risk group (Figure 8F), while the riskscore of dMMR status was significantly lower than that of pMMR status (Figure 8G).

### Prediction analysis of the therapeutic response

We predicted the IC50 value of four common chemotherapeutic drugs (cisplatin, bleomycin, gemcitabine, and etoposide) in the high-risk group and low-risk group of the TCGA dataset. The results showed that the four chemotherapeutic drugs had higher sensitivity in the low-risk group than in the high-risk group (Supplementary Figure S5A). Then, we obtained gene expression data and therapeutic response information of bevacizumab treatment, FOLFIRI treatment, FOLFOX treatment, and methotrexate treatment from GSE19862, GSE62080, GSE28702, and GSE16066 datasets. The results showed that CRC patients sensitive to bevacizumab and methotrexate had lower riskscore, while CRC patients sensitive to FOLFIRI and FOLFOX had higher riskscore (Supplementary Figure S5B). Therefore, we believe that the novel signature can better predict the response of CRC individuals to therapeutic response and provide some guidance for drug selection of CRC patients.

### Functional enrichment analysis in the high-risk and low-risk groups and screening of candidate drugs

**FIGURE 9:**
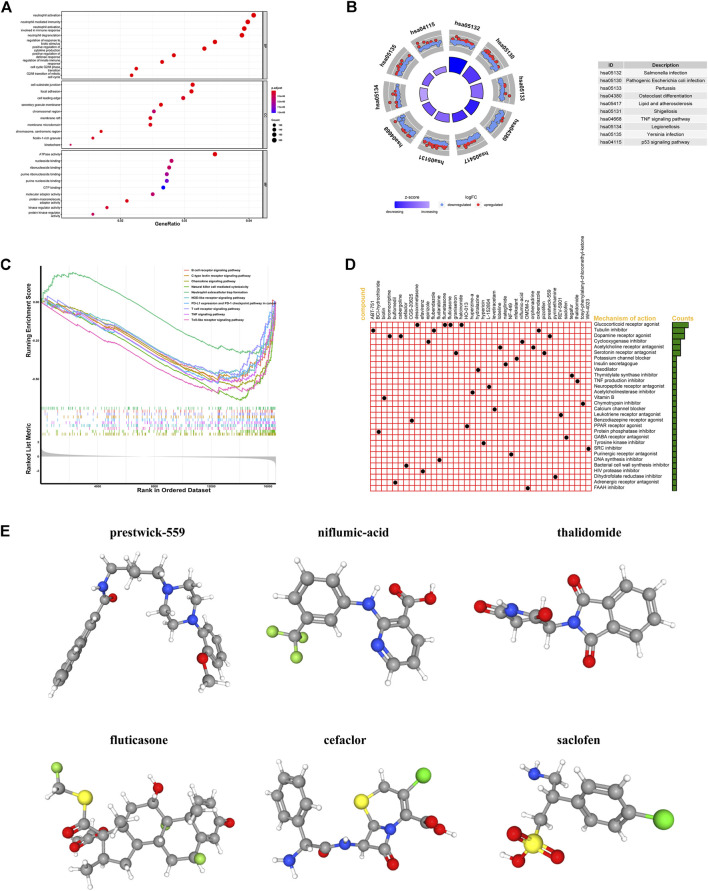
Functional analysis of differentially expressed genes (DEGs) between the high-risk and low-risk groups and screening for small molecule drug. GO, KEGG, GSEA analysis of DEGs between the high-risk and low-risk groups in the TCGA **(A–C)**; **(D)** The mechanisms of action of the top 40 small molecule drugs; **(E)** 3D structures of the top six small molecule drugs.

In order to understand the biological functions and signal pathways related to the IR-lncRNA signature, we performed GO and KEGG analysis on DEGs between the high-risk group and low-risk group of the TCGA dataset. Go enrichment results showed that DEGs are enriched in the signal pathways related to immune response such as neutrophil activation, neutrophil mediated immunity and neutrophil activation involved in immune response, and DEGs are enriched in the molecular function of immune-related molecules such as ATPase activity, nucleoside binding and ribonucleoside binding (Figure 9A). KEGG pathway analysis showed that DEGs were mainly enriched in *Salmonella* infection, Pathogenic *Escherichia coli* infection, TNF signaling pathway and p53 signaling pathway (Figure 9B). GSEA analysis between the high-risk group and low-risk group of the TCGA dataset showed that immune-related signal pathways such as B cell receptor signaling pathway, C-type lectin receptor signaling pathway, Chemokine signaling pathway and Natural-killer-cell-mediated cytotoxicity were significantly enriched (Figure 9C). The up-regulated and the down-regulated DEGs in the high-risk group were uploaded into the CMap database to predict related small molecule drugs, top 40 small molecule drugs were selected based on enrichment score (Supplementary Table S1). We also found that these potential small molecule drugs may act through mechanisms of action such as glucocorticoid receptor agonist, tubulin inhibitor, dopamine receptor agonist, cyclooxygenase inhibitor, acetylcholine receptor antagonist and serotonin receptor antagonist (Figure 9D). The 3D structures of the top six small molecule drugs were obtain from PubChem database (Figure 9E).

### Validation of the expression levels of the six IR-lncRNAs in UALCAN database and CRC clinical specimens by qRT-PCR

**FIGURE 10:**
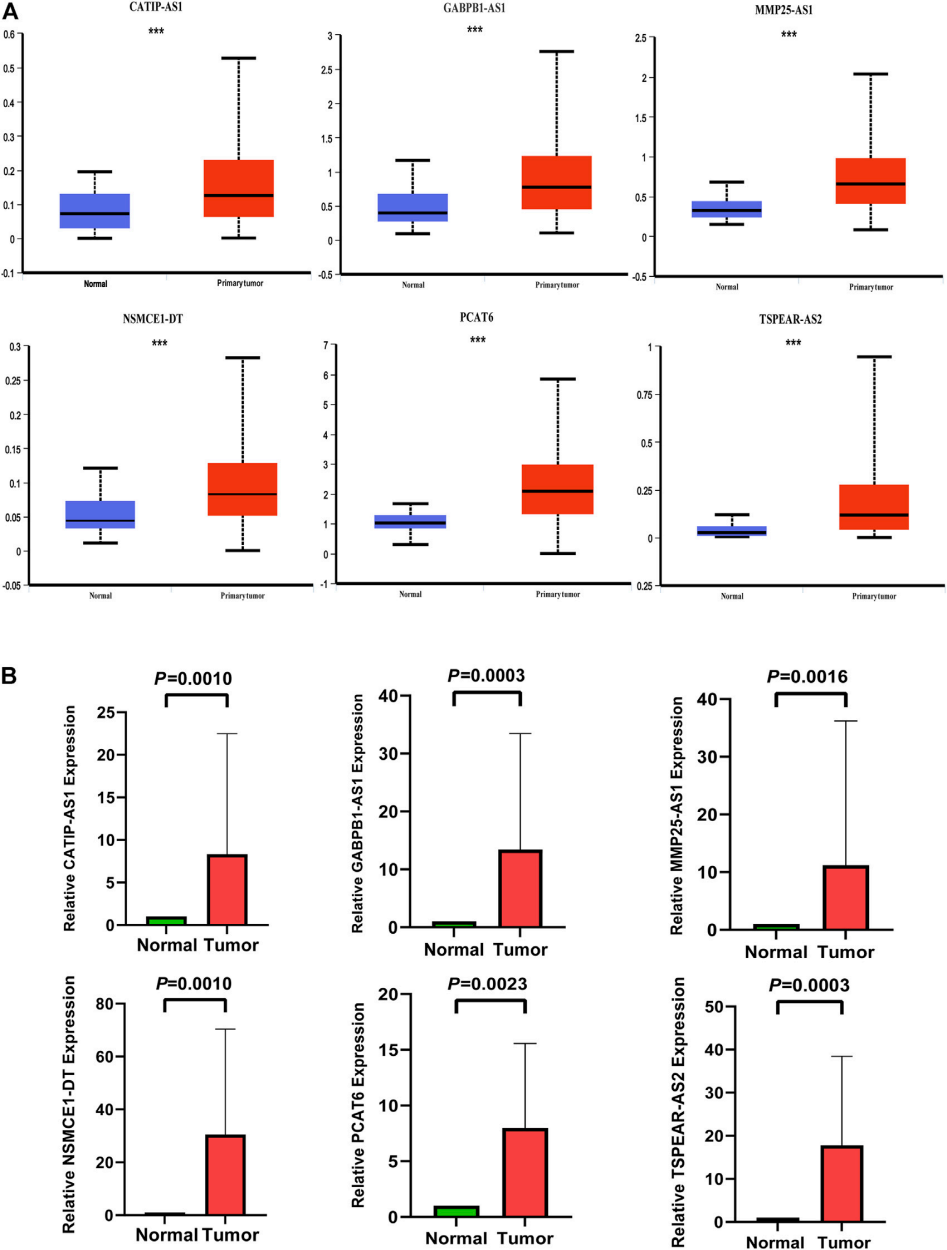
Expression validation of six IR-lncRNAs in CRC clinical specimens by qRT-PCR and UALCAN database. **(A)** Expression validation of six IR-lncRNAs in UALCAN database; **(B)** Expression validation of six IR-lncRNAs in CRC clinical specimens by qRT-PCR. ^*^ *p* < 0.05, ^**^ *p* < 0.01, ^***^ *p* < 0.001. Ns, no significance.

We verified the expression of six IR-lncRNAs in normal and tumor tissues of the TCGA-COAD dataset in UALCAN database (Figure 10A). We collected 36 matched clinical specimens from Ruijin Hospital, Shanghai Jiao Tong University School of Medicine to verify the expression of six IR-lncRNAs by qRT-PCR. The results showed that CATIP-AS1, GABPB1-AS1, MMP25-AS1, PCAT6, NSMCE1-DT and TSPEAR-AS2 were highly expressed in CRC tissues (Figure 10B).

## Discussion

Colorectal cancer is one of the most common malignant tumors, with a high incidence rate and mortality rate. Distant metastasis is the main cause of colorectal cancer death (Malki et al., 2020). It has been reported that the 5-years survival rate of patients with local colorectal cancer is about 90%. Once metastasis occurs, the survival rate decreases rapidly (Osumi et al., 2019), and the mortality of patients aged ≤50 years is 13% higher than that of patients aged >50 years (Siegel et al., 2017). In addition, research shows that more than half of colorectal cancer patients will have distant metastasis, and about 20% of patients will have distant metastasis at the first diagnosis. Immunotherapy combined with chemotherapy, radiotherapy and targeted therapy can significantly improve the curative effect and benefit of patients (Micheal et al., 2018; Lehrer et al., 2019). Therefore, accurate prognosis and active treatment are very important to improve the survival rate of colorectal cancer patients. Traditional TNM staging and tumor biomarkers such as CA125, CA199 and CEA are the main basis for guiding treatment and predicting the prognosis of cancer patients (Jin et al., 2019). However, because the clinical characteristics, therapeutic response, and prognosis of CRC patients are also affected by many factors, such as epigenetic status and microenvironment leading to CRC heterogeneity, traditional TNM staging and tumor biomarkers are difficult to accurately evaluate the prognosis of CRC patients and guide individualized treatment, which cannot meet the actual clinical needs (Linnekamp et al., 2015). Therefore, the development of effective biomarkers to predict the prognosis of CRC patients and the construction of clinical prediction models are of great significance for clinicians to accurately predict the survival status of CRC patients, guide clinical individualized treatment and improve the survival rate of patients.

In this study, we screened the six IR-lncRNAs (CATIP-AS1, GABPB1-AS1, PCAT6, MMP25-AS1, NSMCE1-DT and TSPEAR-AS2) from the public database and used them to construct a prognostic model for predicting the survival of CRC patients. Interestingly, the analysis results of the TCGA training dataset and GSE39582 validation dataset show that the novel signature is not only an independent predictor of the prognosis of CRC patients but also can accurately predict the tumor immune microenvironment features and therapeutic response of CRC individuals. The name of CATIP-AS1 is CATIP antisense RNA 1. At present, the research about CATIP-AS1 in CRC has not been reported, only Rao et al. found for the first time that the down-regulation of CATIP-AS1 is related to the longer disease-free survival time of patients with thyroid cancer (Rao et al., 2020). The name of GABPB1-AS1 is GABPB1 antisense RNA 1, Ou et al. reported that the high expression of GABPB1-AS1 is associated with the poor prognosis of HPV16 positive cervical cancer patients. GABPB1-AS1 can release the inhibition of its target gene Notch2 and promote the progress of cervical cancer by binding with miR-519e-5p (Ou et al., 2020). In gliomas, the high expression of GABPB1-AS1 can lead to the activation of cell cycle signal pathway and the progression of glioma cells (Li and Wang, 2021), while Qi et al. found that the high expression of GABPB1-AS1 is related to the improvement of overall survival of patients with hepatocellular carcinoma and can inhibit the antioxidant capacity of tumor cells (Qi et al., 2019). The name of PCAT6 is prostate cancer-associated transcript 6. There are many studies on the mechanism of PCAT6 in various types of malignant tumors. Huang et al. found that PCAT6 is up-regulated in colon cancer tissue, which is related to poor survival status. PCAT6 can inhibit apoptosis and promote the progress of colon cancer by forming a complex with EZH2 (Huang et al., 2019). A study of bladder cancer showed that overexpression of PCAT6 can promote the progression of bladder cancer by targeting miR-513a-5p (Xia et al., 2020). The name of MMP25-AS1 is MMP25 antisense RNA 1. At present, the research on MMP25-AS1 is only reported in renal cell carcinoma. Tan et al. found that the high expression of MMP25-AS1 was significantly correlated with the poor survival of patients with early and advanced diseases. MMP25-AS1/hsa-miR-10a-5p/SERPINE1 axis may be a novel mechanism to promote the progression of renal clear cell carcinoma (Tan et al., 2021). The name of NSMCE1-DT is NSMCE1 divergent transcript. At present, there is no relevant research. This is the first time that we propose that the high expression of NSMCE1-DT may be a risk factor for the prognosis of CRC. The name of TSPEAR-AS2 is TSPEAR antisense RNA 2. There are still few studies on TSPEAR-AS2. Ma et al. found that TSPEAR-AS2 can promote the progression of gastric cancer by inhibiting the expression of GJA1 and up-regulating the expression of CLDN4 (Ma et al., 2020). In CRC, only Peng et al. found that the high expression of TSPEAR-AS2 could lead to poor prognosis in CRC patients (Peng et al., 2021). Finally, the expression of six IR-lncRNAs were verified in CRC and normal clinical specimens by qRT-PCR.

Immunity is an important life activity to maintain the homeostasis of the internal environment. It has the function of immune monitoring, defense and regulation. Studies have shown that the state of immune microenvironment is not only an important biological feature of tumor, but also an important factor in prognosis. Immune escape, immune activation and tumor-promoting inflammation have become new tumor markers (Khalaf et al., 2021). Various components of the immune system involved in the pathogenesis and development of tumors have been proved to be the key factors of tumors. As an important regulatory factor in the immune system, lncRNA plays an important role in the pathogenesis and development of tumors. However, there are few studies on how lncRNA regulates tumor immune microenvironment and immune cell infiltration. Huang et al. found that inhibiting lncRNA NKILA can regulate the tumor microenvironment by reducing T cell apoptosis and enhancing its killing ability (Huang et al., 2018). An analysis of single-cell sequencing data of liver cancer immune cells found that lncRNA MIAT was significantly enriched in Foxp3^+^ CD4^+^ T cells, PDCD1^+^ CD8^+^ and GZMK^+^ CD8^+^ T cells, which mediated the immune escape of liver cancer (Peng et al., 2020). Zhou et al. found that the expression of lncRNA SNHG4 was up-regulated in CRC tissue. Through targeted down-regulation of miR-144-3p, it induced CD4^+^ T cell apoptosis and activated PD-1/PD-L1, which mediated the immune escape of colorectal cancer (Ning et al., 2021). In this study, the features of immune microenvironment in high-risk and low-risk groups based on the IR-lncRNA signature were significantly different. Compared with the low-risk group, the high-risk group had higher tumor purity, lower immune score and cytolytic activity, and the high-risk group had more immunosuppressive cell infiltration and less immune-activated cell infiltration in the microenvironment. The immune microenvironment of the high-risk group mainly showed immunosuppression, while the low-risk group mainly showed immune activation. Correlation analysis showed that the expression of CATIP-AS1 was positively correlated with the infiltration of plasma cells, the expression of NSMCE1-DT was negatively correlated with the infiltration of CD8^+^ T cells, the expression of PCAT6 was positively correlated with the infiltration of plasma cells, and negatively correlated with the infiltration of M1 macrophages and CD4^+^ memory activated T cells. Thus, lncRNA plays an important role in the regulation of tumor immune microenvironment.

Immunotherapy is a new milestone in tumor therapy. It has become another effective means after surgery, radiotherapy, chemotherapy and targeted therapy. Keynote-177 clinical study found that the median PFS (16.5 months) of pembrolizumab single drug first-line treatment was twice that of the control chemotherapy group (8.2 months) (HR = 0.60; 95% CI: 0.45–0.80; *p* = 0.0002), while the objective response rate (ORR) of pembrolizumab treatment group was only 43.8% and that of chemotherapy group was 33.1% (Andre et al., 2020). The checkmate-142 clinical study found that the ORR and disease control rate (DCR) of patients treated with Ipilimumab and nivolumab were 69% (95% CI: 53-82) and 84% (95% CI: 70.5-93.5), respectively, while the complete remission rate was only 13% (Hein-Josef et al., 2022). Therefore, immunotherapy improves the prognosis of CRC patients to a certain extent and is a promising treatment option. However, at present, only some CRC subtypes have good immune response to immunotherapy. Therefore, in the future, it is necessary to further explore effective biomarkers, further accurately stratify CRC patients, find out more accurate immunotherapy dominant groups, and select the optimal treatment scheme to benefit more CRC patients. In this study, compared with the high-risk group, tumor immune checkpoint (IC) is highly expressed in the low-risk group, suggesting that patients in the low-risk group may have a better response to immune checkpoint inhibitor treatment. A large number of studies have shown that high TMB and MSI-H/dMMR are effective tumor markers for immunotherapy. In this study, compared with the high-risk group, patients in the low-risk group have higher TMB and more MSI-H/dMMR subtypes, which further indicates that the low-risk group may be more sensitive to immunotherapy and benefit from immunotherapy. Therefore, the above results show that the IR-lncRNA signature in this study can well predict the sensitivity of CRC patients to immunotherapy and provide guidance for personalized treatment.

Based on the differentially expressed genes in the high-risk and low-risk groups, we further explored and identified new strategies for the treatment of CRC. We used the CMap database to screen for 40 small molecule drugs that could potentially reverse and treat high-risk patients. These potential small molecule drugs may act through mechanisms of action such as glucocorticoid receptor agonist, tubulin inhibitor, dopamine receptor agonist, cyclooxygenase inhibitor, acetylcholine receptor antagonist and serotonin receptor antagonist. This not only provides a new solution to the problem of poor prognosis in high-risk CRC patients, but also lays a theoretical foundation for further drug development.

In conclusion, by screening immune related lncRNAs, we finally constructed a novel IR-lncRNA signature that can accurately predict the prognosis, immune microenvironment features and therapeutic response of CRC patients. Although there have been studies on the prediction of prognosis by IR-lncRNAs in colorectal cancer, our study still has the following advantages. Firstly, we systematically screened IR-lncRNAs, constructed and verified a novel IR-lncRNA signature that can accurately predict the prognosis of CRC patients for the first time by using TCGA dataset and GSE39582 dataset. Secondly, we found that this IR-lncRNA signature can not only accurately predict the prognosis of CRC patients, but also has potential clinical value in the features of immune microenvironment and therapeutic response. Then, the six IR-lncRNAs were highly expressed in CRC patients and verified by qRT-PCR in our own clinical samples. NSMCE1-DT, CATIP-AS1, GABPB1-AS1 and MMP25-AS1 are the risk factors for the prognosis of CRC patients that we found for the first time. Among them, NSMCE1-DT has not been reported in various malignant tumors, which is worthy of further study on its function and mechanism. Finally, this novel signature may also have good predictive value for the prognosis and immune microenvironment characteristics of other tumors, but it needs to be further verified. At the same time, our research also has limitations. Since we used the retrospective data of the public database as the training dataset and validation dataset, a prospective, multicenter clinical cohort may be needed in the future to further verify its clinical predictive value and its relationship with the clinical characteristics of CRC patients. In addition, the function and specific mechanisms of these six IR-lncRNAs in CRC deserve further exploration in order to better understand the role of lncRNA in the pathogenesis and development mechanism of CRC.

## Conclusion

In conclusion, we constructed and verified a reliable and effective IR-lncRNA prognostic signature, which can not only accurately predict the prognosis of CRC patients, but also effectively predict the immune microenvironment features and therapeutic response of CRC individuals. This novel IR-lncRNA signature provides a new idea for the prognosis stratification and individualized treatment management of CRC, or will become a newly accurate prediction tool to evaluate the prognosis of CRC patients and therapeutic response. At the same time, it also lays a foundation for understanding the regulatory role of lncRNAs in the tumor immune microenvironment. Finally, we screened small molecule compounds that could be used to treat and reverse the high-risk patients based on the DEGs between high-risk and low-risk patients.

## Data Availability

The datasets presented in this study can be found in online repositories. The names of the repository/repositories and accession number(s) can be found in the article/Supplementary Material.

## Abbreviations

CRC, Colorectal cancer; IR-lncRNA, Immune-related lncRNA; LASSO, the Least Absolute Shrinkage and Selection Operator; TME, Tumor microenvironment; DEG, Differentially expressed gene; IC, Immune checkpoint.
