# Supplementary figures and images for: Comprehensive exploration of tumor immune microenvironment feature and therapeutic response in colorectal cancer based on a novel immune-related long non-coding RNA prognostic signature
Source: Front Genet. 2022 Aug 25;13:962575. doi: 10.3389/fgene.2022.962575 (PMC9454821; doi:10.3389/fgene.2022.962575)

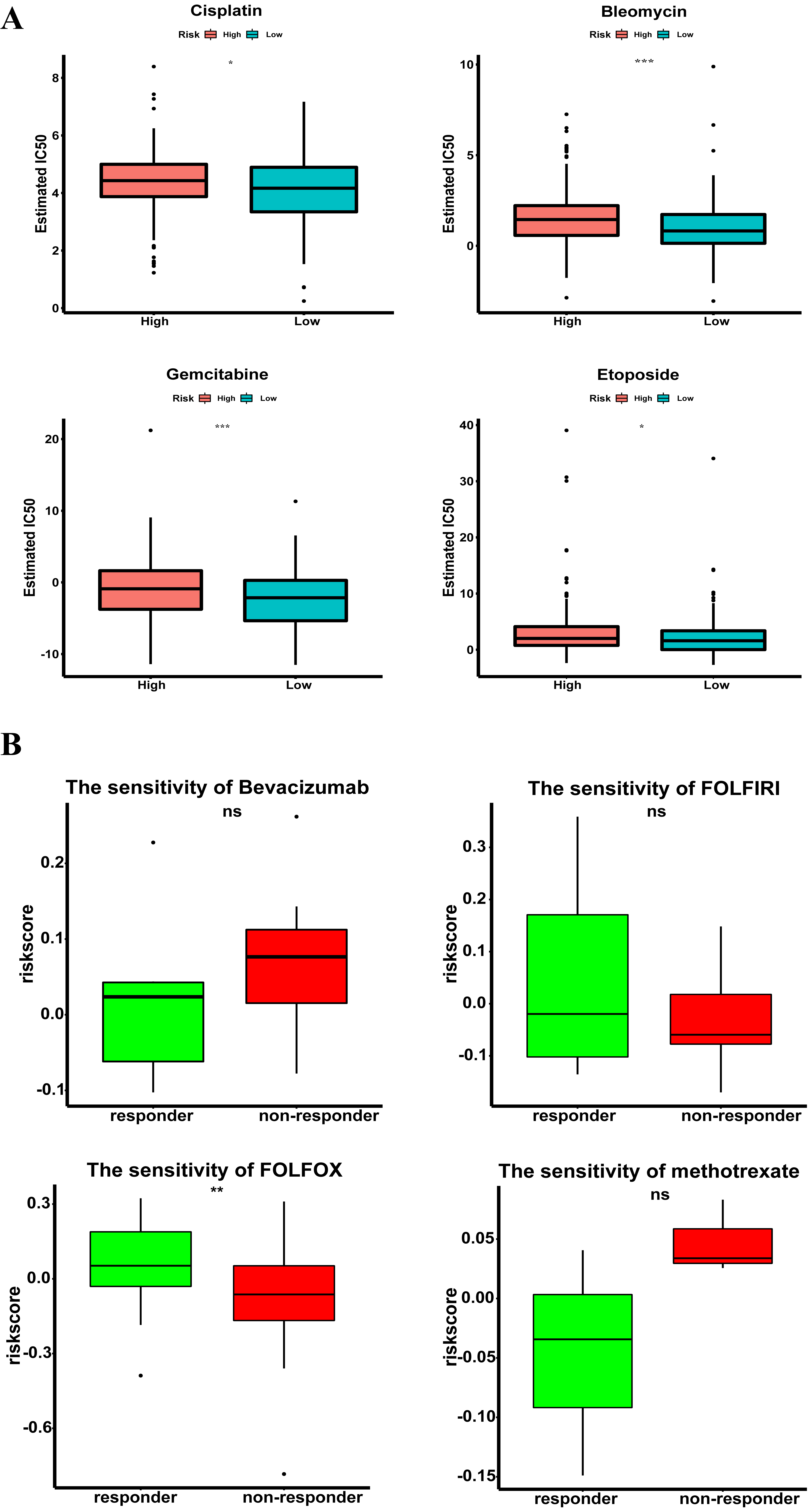

Supplement: Supplementary file 1 [file DataSheet1.ZIP › Figure-S5.tif]
